# Inverse design of adaptive flexible structures using physical-enhanced neural network

**DOI:** 10.1080/17452759.2025.2530732

**Published:** 2025-07-18

**Authors:** Moslem Mohammadi, Abbas Z. Kouzani, Mahdi Bodaghi, Ali Zolfagharian

**Affiliations:** a School of Engineering, Deakin University, Geelong, VIC, Australia; b Department of Engineering, School of Science and Technology, Nottingham Trent University, Nottingham, UK

**Keywords:** Metamaterials, buckling, flexible structures, soft robotics, 3D printing

## Abstract

Traditional design and analysis of mechanical metamaterials are complex and time-consuming, owing to their nonlinear characteristics. This paper proposes a computationally efficient inverse design framework to predict the nonlinear strain–stress response considering the buckling behaviour under a tensile load. Design and simulation processes of the structures are based on the reduced order model (ROM) of flexible structures, all within a single software environment, MATLAB/Simscape, using the flexible beam blocks. The physical-enhanced neural network (PENN) design is implemented in MATLAB, utilising the results of the ROM model for training and testing. The ROM model takes 4.5 min on average on a 12-core CPU, whereas the trained PENN predicts the stiffness curve in a fraction of a second on a single-core CPU. After training the model, it was utilised to inverse design the metamaterial structure based on a desired stiffness response. Evolutionary optimisation is employed to iteratively feed various structural parameters into the model to find the optimised parameters of a metamaterial structure that can achieve the desired strain–stress response. The proposed metamaterial structure was experimentally validated through three-dimensional (3D) printing using flexible thermoplastic polyurethane (TPU) filament, demonstrating the efficiency and effectiveness of the proposed methodology.

## GRAPHICAL ABSTRACT

**Figure:**
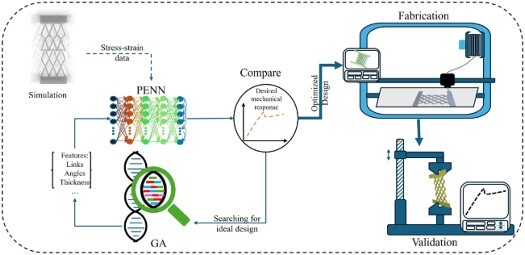

## 1. Introduction

Researchers use metamaterials to solve several challenges with creating mechanical structures, such as designing vibration-damping mechanisms, reducing the weight, attenuating energy/shock, designing auxetic structures, miniaturising mechanical structures, and devising tuneable mechanical properties [1–4]. The diverse mechanical reactions of metamaterials, which are engineered structures designed to present properties that are not inherited from the base material but from the specific structural/geometrical configurations, and its elements are usually arranged in a repeating pattern, can be utilised to overcome these obstacles [5]. Depending on the external stimuli, they can transition between elastic-dominated and damping-dominated regimes with variable average stiffness under dynamic loading [2]. Additionally, they provide a wide range of mechanical characteristics through the recombination of fundamental materials and configuration [6]. The potential advantages of metamaterials, specifically their non-linear characteristics, are significant. In the context of electromagnetic fields, for instance, the engineered geometric structure can produce exceptional phase-matching conditions and substantial local field amplification in order to optimise a specified nonlinear mechanism [7]. The growth and advancement of computational methods have facilitated enhanced exploration of the realm of potential configurations and more potent strategies for the logical design of metamaterials [5] (Table 1).

**Table 1.** Table of abbreviations.

Abbreviation	Definition
ROM	reduced order model
PENN	physical-enhanced neural network
3D	three-dimensional
TPU	thermoplastic polyurethane
AM	additive manufacturing
FEA	finite element analysis
2D	two-dimensional
FE	finite-element
MAE	mean absolute error
RMSE	root mean squared error
ReLU	rectified linear unit
SGDM	stochastic gradient descent with momentum
NN	neural network

Mechanical Metamaterials characteristics come from their engineered structures rather than the original materials themselves [8–11]. Modifying the geometry and form of these structures, like quadrilateral cell shapes, allows for the implementation of a wide variety of nonlinear mechanical reactions [12]. Metamaterial structure design and fabrication are intricate processes. The design methods of metamaterials have been expanded, and additive manufacturing (AM) techniques, also known as three-dimensional (3D) printing, have facilitated the fabrication of those complex structures [5]. They facilitate the generation of structures and materials of diverse length scales and materials, exhibiting unprecedented complexity in their distributions within a single process [5]. This methodology has been used in the fabrication of intricate structures with a desired mechanical property. Design methods involve a range of techniques for optimising mechanical response [13], topology [14], and shape [15], with periodic and aperiodic [16] structures, based on various models including ML algorithms [17]. In order to construct metamaterial structures with nonlinear responses, ML approaches have been reported [18,19]. However, these approaches have yet to be implemented in mechanical metamaterials characterised by variable sizes, and a broad variety of nonlinear responses, particularly with the capability of bucking or snapping through behaviour consideration. This design deficiency provides a potential path for more investigation and advancement in the design of metamaterials.

To get the required response by direct design of a metamaterial structure, which is a highly nonlinear problem and almost impossible to locate the optimal point in traditional ways, several solutions based on optimisation algorithms have been proposed, such as particle swarm optimisation [20,21] and other evolution strategies [19,22]. In some instances, these algorithms must be iterated hundreds of times; hence, system simulations and experiments are quite costly. Researchers have effectively applied various machine learning methods to enhance accuracy and efficiency when designing mechanical metamaterials inversely. In one study, Zeng et al. developed a deep neural network architecture trained by topology optimisation generated dataset, allowing for fast and very precise predictions [23]. Abu-Mualla and Huang created a neural network model that demonstrated superior computational efficiency and prediction accuracy over traditional methods [24]. Hussain et al. proposed an artificial neural network model to represent the larger-scale mechanical behaviour of metamaterials within a three-dimensional space [25]. Deng et al. combined an artificial neural network with the whale optimisation algorithm for the inverse design of metamaterials exhibiting nonlinear stress–strain behaviours, achieving high performance with an R2 value of 0.981 [26]. Recent work has shown the potential of swarm-based algorithms such as grey wolf optimisation for improving mechanical performance in fused deposition modelling processes through rapid convergence and effective parameter search [27]. Alderete et al. employed two deep neural networks to predict deformations based on ABAQUS simulations for the inverse design of kirigami metamaterials and predicting metamaterials deformation [28]. Pahlavani et al. focused on multi-material metamaterials using machine learning models for inverse design [29]. Soo Ha et al. utilised a deep neural network to inversely design lattice structures based on desired stress–strain curves, achieving 90% similarity between intended and experimental results using 3D printing [30]. Even though much significant research has been done recently on the inverse design of metamaterials based on ML algorithms [19,22], knowledge gaps exist in the design and analysis of these structures that are addressed in this research. Design methods based on finite element analysis (FEA) and ROM are quicker than experiments, but they are still complex and time-consuming. While these methods have demonstrated success, they often demand extensive computational resources.

This work is motivated by the need for faster, more efficient inverse design of nonlinear mechanical metamaterials, especially those exhibiting complex behaviours such as buckling. A significant challenge in this work is predicting the buckling effect in the stiffness curve of metamaterials using machine learning models, which is effectively tackled by the proposed approach. Furthermore, the model automates the optimisation and fabrication processes through AM and reduces computational resources with the use of PENN. A novel loss function is introduced to enhance neural network performance and improve training efficiency. The input data for the neural network is derived from a down-sampled stiffness curve, which reduces the number of parameters in the machine learning model, thus decreasing calculation costs and time.

The key novel aspects of this study are: (1) a physics-enhanced neural network (PENN) trained on ROM data; (2) a customised loss function for capturing buckling; and (3) an integrated design-to-fabrication pipeline using GA and 3D printing. The significance of this research lies in its potential to substantially decrease the time and computational resources required for the inverse design of metamaterial samples and the customisation of the buckling effect under tensile force for various applications. Traditional neural networks rely solely on data to identify patterns and use conventional loss functions to measure discrepancies between the model’s output and the labels. In contrast, this research integrates related physics knowledge and insights into the learning process, model architecture, and loss function of the machine learning model. In PENN, the stiffness curve is predicted to exhibit spatial dependencies based on the mechanical structure’s stiffness response. This dependency suggests that each value on the curve is influenced by preceding values and the rate of change along the curve. This physical rule is incorporated into the last layer of the neural network, and the loss function is modified to ensure that the values of the stiffness curve follow an increasing trend. This modification helps the model train more efficiently and results in higher precision compared to traditional deep neural networks, especially in predicting the buckling effect in the stiffness curve. Additionally, the study is further validated through experimental testing of the strain–stress response of the inverse-designed structure using AM with TPU filament, ensuring the practical applicability of the proposed design paradigm.

**Figure 1.**
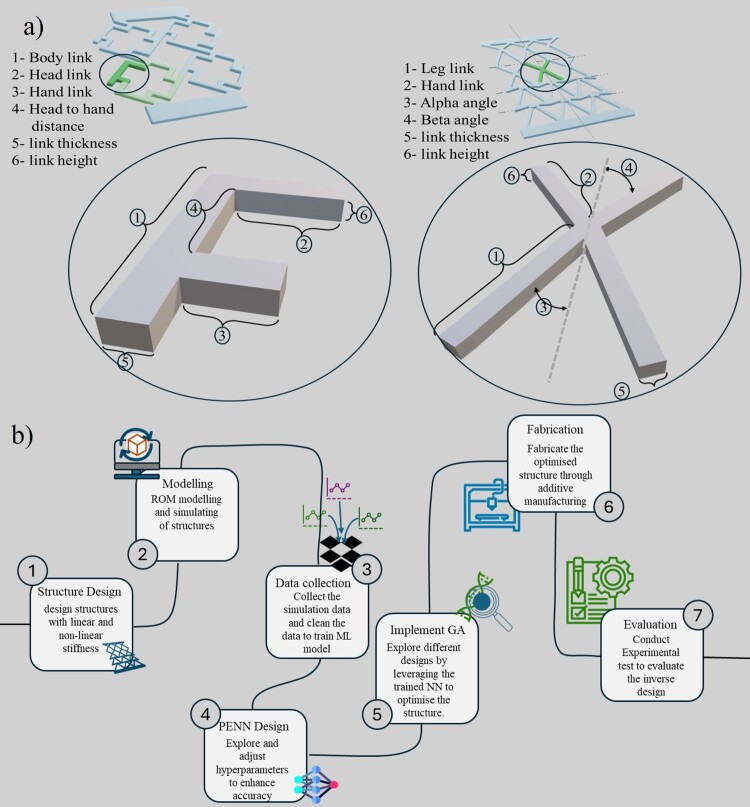
(a) Various cells of the framework and their variable parameter. (b) The workflow of inverse design of metamaterials.

This study focuses on parameterised bistable and linear unit-cell designs simulated with ROM, modelled using a PENN, and optimised through genetic algorithms, with validation via 3D printing. A methodology is presented for the design and fabrication of metamaterials in this article, demonstrating a wide range of nonlinear strain–stress responses. Our structures’ base cells are illustrated in Figure 1(a) (each cell is structured as either an F-shape or an X-shape entity), which can have variable stiffness on different scales. Furthermore, the Poisson ratio of the structure may display a variety of ranges depending on the various parameters taken into account. Mathematical models and simulation findings allow us to first show that different mechanical responses are produced by different configurations of the cell components. After that, we use PENN to find the exact relationship between the geometry of the metamaterials and their stress–strain parameters. In the end, we find the geometries of a structure with the appropriate nonlinear stress–strain properties by combining the trained PENN with an optimisation technique. Designing systems for energy and shock absorption, flexible electronics, and robotics are just a few examples of the numerous possible applications of the suggested method, which is based on ML models. Afterwards, the structure is fabricated through additive manufacturing. The structure’s behaviour is evaluated via experimental tests (Figure 1b). Unlike topology optimisation, which focuses on free-form material distribution, our method uses a parameterised design space of snapping-enabled unit cells to efficiently predict and optimise nonlinear stiffness responses. The F – and X-unit frameworks were chosen to validate the inverse design workflow using physically enhanced neural networks. While the number of unit cells and parameters is limited compared to full-scale topology optimisation, this setup effectively demonstrates the feasibility and robustness of the proposed method for linear and nonlinear response design.

## 2. Methodology

The design and fabrication of non-linear stiffness structures by considering two different structures are depicted in Figure 1. Both structures include 48 flexible links, but with different configurations. In Figure 1(a), the structure is made by repeating a cell four times which is highlighted in light green. The mentioned cell is shaped by an F-unit structure four times, and they are rotated 90 degrees compared to each other. This F-unit cell has 6 parameters, including the length of its links and the links’ thickness and height. By changing these parameters, we are able to obtain a broad range of structures that will provide various stiffnesses. The length of the body link can change from 10 mm to 40 mm, and the head and hand links’ length range from 5 to 40 mm. The minimum value for the head of the structure to its hand distance is 5 mm, which is more than the maximum value for link thickness to avoid any collision between the head and hand links. The maximum value for that distance is the length of the body link to cover the full range. Also, all the links’ thicknesses and heights are between 1–5 mm.

**Figure 2.**
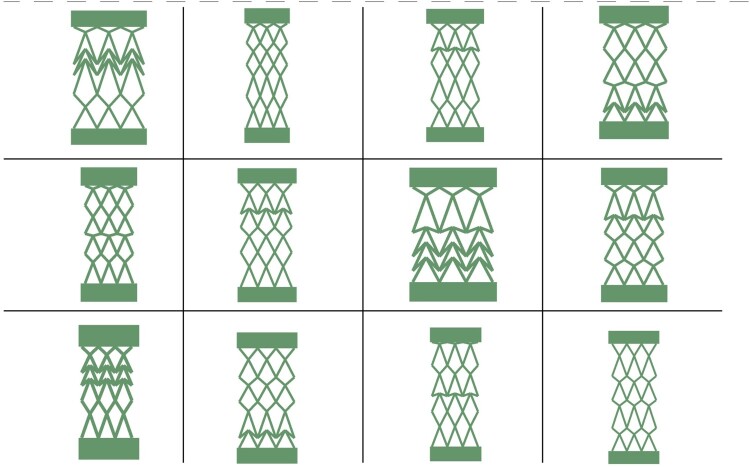
Various configurations of X-unit structures.

The F-unit cell can show various vlaues of Poisson’s ratio, including positive and negative values and approximately linear stiffness (fabricated with linear stiffness material). On the other hand, the X-unit structure shows linear and nonlinear force versus displacement based on the configuration of the links. It can also perform as a positive or negative Poisson ratio metamaterial. This simple structure is fabricated based on 12 X-unit cells that are arranged in 4 rows. In each row, the cells are identical, but they have different configurations compared to the adjacent rows. The X-unit cell has six parameters to be set, including its leg link, hand link, their deviation from the axis of symmetry, and the links’ thickness and height. The deviation of the leg links (alpha angle) of the cell can vary from $\frac{\pi }{10}$ to $\frac{\pi }{6}$ and beta angle. The length of the leg link can change from 20 to 40 mm. The minimum value for the hand link is the long link length multiplied by the sinus angle of alpha, and its maximum is 0.8 of the leg length. The beta angle can be calculated depending on the other parameter, but still, it can be an acute or obtuse angle randomly as the width of the cell must remain the same due to manufacturing constraints. These values and their limits are chosen based on the mechanical constraints (avoid overlapping of the beams) of the structure and the constraints of the fabrication process, including the size and capabilities of the 3D printer. Figure 2(b) depicts different configurations of this structure in 12 different formats as examples, while retaining key details.

These geometries are selected intentionally because of their varied applications and complementing mechanical characteristics. The ‘F’ shape is perfect for applications needing constant resistance to deformation because of its linear stiffness response, which provides predictable and stable performance. The ‘X’ form, on the other hand, exhibits nonlinear mechanical reactions due to geometric nonlinearities, such as large deformations and buckling. These elastic nonlinearities, which are distinct from material effects like plasticity or hysteresis, stemming instead from the structural configuration, are essential for adaptive systems like shock isolation, vibration dampening, and energy absorption. Due to their adjustable link lengths, thicknesses, and angles, both unit cells offer a great deal of design freedom and allow for exact control over stiffness and Poisson's ratio. Configurations with positive or negative Poisson's ratios and customised mechanical responses for a range of applications are made possible by this adaptability. The ‘F’ and ‘X’ forms’ opposing characteristics, which handle both linear and nonlinear responses, verify the approach. Furthermore, their simple yet adaptable designs offer excellent experimental validation through practical 3D printing and reduced-order modelling (ROM) simulations.

### 2.1. Mathematical model

To determine the stiffness of a structure composed of interconnected flexible components, we can use assumptions to build mathematical models. Each flexible component in the structure is assumed to follow Hooke's law, where deformation is directly proportional to the applied load within the material's elastic range. The links are assumed to have a slender form, so deformation from bending rather than stretching or compression is primarily considered, allowing simplification by focusing solely on bending deformation. The structure is assumed to lie within a two-dimensional (2D) plane, simplifying the analysis to a 2D problem. Here are the steps to develop a mathematical model for determining the structure's stiffness. The structure's geometric details are specified (link sizes, connection nodes), which is shown in Figure 1, along with the material properties of the flexible components, TPU (Young's modulus and density). Then equilibrium principles are applied at each node to generate equations balancing forces and moments. The boundary conditions like fixed or pinned link connections are also defined to determine system constraints. While structural design plays a crucial role in determining the behaviour of metamaterials, the performance is significantly influenced by the intrinsic properties of the base materials. Properties such as Young’s modulus, damping coefficient, and strain-rate sensitivity directly affect how the structure responds to dynamic or large-strain loading. A well-tuned synergy between structural geometry and material choice enables metamaterials to achieve tailored stiffness, controlled energy absorption, and even auxetic responses. This study focuses on TPU due to its elasticity and printability, which are well-suited for capturing snap-through and bistable behaviour.

Here, we develop the mathematical equation for a general structure, which is the basis of Simscape calculation for flexible bodies in MATLAB software. The Simscape calculation of flexible bodies is based on the ROM, which is calculated based on the finite-element (FE) model, which divides the body into a polygonal mesh with many nodes (Supplementary Figure 1), each having up to six degrees of freedom. All FEM operations used default settings provided by MATLAB Simscape’s flexible body environment, including mesh density and integration schemes, to ensure consistency and stability across simulations. The mesh is governed by the equation of motion [31,32].

(1) $$M{\ddot{u}}_{d}+L{\dot{u}}_{d}+Ku_{d}=f$$

The matrix **M** represents the discretised body’s mass and matrices **L** and **K** refer to its damping and stiffness, respectively. The nodal degrees of freedom are shown by $u_{d}$ and f is the array of the external loads at each node of the body. The second-order differential equation is replaced with a system of first-order equations known as the state-space representation. The following equation is our state equation:

(2) $$\dot{x}=\mathrm{Ax}+\mathrm{Bu}$$

Our output equation is:

(3) y=Cx+Du

where **x** represents the state vector, and **u** and **y** are input and output. Also, the state-space representation matrices are defined as **A** (state matrix), **B** (input matrix), **C** (output matrix), and **D** (direct feedthrough matrix). The state-space representation matrices **A**, **B**, **C**, and **D** are determined using the mass, damping, and stiffness matrices derived from the FE model and calculated as follows [31,32]:

$$A=\left[\begin{array}{cc} O & I\\ -{\hat{M}}_{\mathrm{mm}}^{-1}{\hat{K}}_{\mathrm{mm}} & -{\hat{M}}_{\mathrm{mm}}^{-1}{\hat{L}}_{\mathrm{mm}} \end{array}\right]$$

$$B=\left[\begin{array}{ccc} O & O & O\\ -{\hat{M}}_{\mathrm{mm}}^{-1}{\hat{K}}_{\mathrm{mb}} & -{\hat{M}}_{\mathrm{mm}}^{-1}{\hat{L}}_{\mathrm{mb}} & -{\hat{M}}_{\mathrm{mm}}^{-1}{\hat{M}}_{\mathrm{mb}} \end{array}\right]$$

$$C=\left[\begin{array}{cc} -({\hat{K}}_{\mathrm{bm}}-{\hat{M}}_{\mathrm{bm}}{\hat{M}}_{\mathrm{mm}}^{-1}{\hat{K}}_{\mathrm{mm}}) & -({\hat{L}}_{\mathrm{bm}}-{\hat{M}}_{\mathrm{bm}}{\hat{M}}_{\mathrm{mm}}^{-1}{\hat{L}}_{\mathrm{mm}}) \end{array}\right]$$

(4) $$\begin{array}{r} D=\left[\begin{array}{ccc} -({\hat{K}}_{\mathrm{bb}}-{\hat{M}}_{\mathrm{bm}}{\hat{M}}_{\mathrm{mm}}^{-1}{\hat{K}}_{\mathrm{mb}}) & -({\hat{L}}_{\mathrm{bb}}-{\hat{M}}_{\mathrm{bm}}{\hat{M}}_{mm}^{-1}{\hat{L}}_{\mathrm{mb}}) & \\ -({\hat{M}}_{\mathrm{bb}}-{\hat{M}}_{\mathrm{bm}}{\hat{M}}_{\mathrm{mm}}^{-1}{\hat{M}}_{\mathrm{mb}}) \end{array}\;\;\;\;\;\;\right] \end{array}$$

The details of the outlined equations are discussed in supplementary document – S1.

In addition to the ROM-based dynamic formulation, we reference classical Euler buckling theory to establish a theoretical baseline for the critical buckling load of individual flexible links. Assuming each link behaves as a slender, axially loaded beam with pinned-pinned boundary conditions, the critical buckling force $P_{\mathrm{cr}}$ can be approximated using the Euler formula [33]:

(5) $$P_{\mathrm{cr}}=\frac{\pi^{2}\mathrm{EI}}{{\left(K_{\mathrm{eff}}L\right)}^{2}\;}$$

where, E is the Young’s modulus of the material (TPU), I is the second moment of area for a rectangular cross-section of width b and height h, L is the effective length of the beam (link), and $K_{\mathrm{eff}}$ is the effective length factor, taken as 1.0 for pinned-pinned conditions. This analytical result serves as a simplified estimate for the onset of buckling, against which the ROM-based deformation behaviour can be qualitatively compared. While the multibody ROM model used in Simscape captures more complex dynamics – such as geometric nonlinearity, snapping, and joint coupling – this classical solution provides useful insight into local buckling behaviour within a single link and validates that the critical loads observed in simulation are within physically realistic bounds.

### 2.2. Simulation

In Simulink, the structures of the mentioned frameworks are simulated mainly based on a general flexible body. The motion equation of the proposed frameworks is based on the outlined equation above. The modelling framework is modular and can be generalised to simulate other structures, assuming the material properties and structural parameters are adapted to the new task. For each link in our frameworks, one single flexible body is imported into our model. The material parameters, including density, Young’s modulus, and Poisson’s ratio, are set based on TPU features. It is important to note that TPU does not have a truly linear Young’s modulus. In this study, a constant value was assumed to reduce computational complexity. This simplification is justified by experimental observations, where the maximum strain in the beams remained below 5%, a range in which TPU exhibits approximately linear behaviour. In this regime, deformation is primarily driven by beam reconfiguration rather than material stretching, making the linear approximation reasonably valid. However, during buckling events, localised strains may exceed this threshold, potentially reducing simulation accuracy. This contributes to simulation-experimental discrepancies, which average 6.8% error, with peaks of 35.7% during buckling regions (Figure 8a). In addition to material simplification, idealised joint modelling further contributes to this deviation. To improve reliability, future models will incorporate hyperelastic material properties and advanced joint representations. Links’ cross section is valued to change their height and thickness, and their length is determined based on the value in the length field. Various joints and transformations are used to form the whole structure. To calculate the framework's stiffness, the framework is stretched through a prismatic joint, and the required force is measured. The stiffness curve is fed to a PENN model to train it, and due to the nonlinearity of the structures’ stiffness, a large amount of data is required to train the model properly.

**Figure 3.**
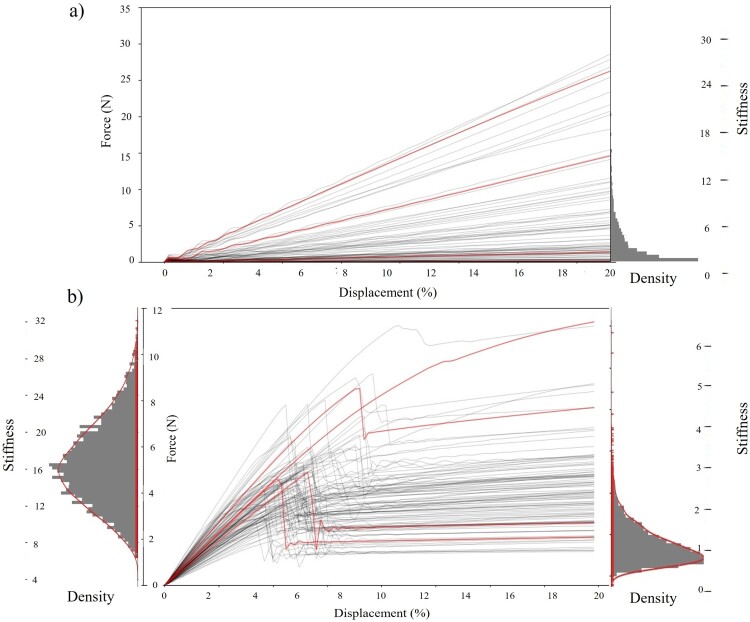
Simulation results and stiffness distribution for (a) F-unit framework and (b) X-unit framework.

The Simulink model is run 5000 times for the X-unit framework and 4000 times for the F-unit metamaterial. We incrementally increased the number of simulations and monitored the PENN’s performance on a separate validation set. Once RMSE plateaued across multiple random seeds, we considered the dataset size sufficient for training. The difference between the numbers is due to the stiffness curve of the structures, which is more complex for the X-unit framework, and to reduce the evaluation and testing error of the PENN model, more data collected on that specific framework. The simulation results of the stiffness curves are depicted in Figure 3. The stiffness of the F-unit structure (Figure 3a) is mainly linear; however, its distribution is not uniform throughout the range. The structure distribution of stiffness for various configurations is depicted on the left side histogram of Figure 3(a). Thus, to collect more curves with higher stiffness, the simulation is run 4000 times, and some of the curves in the lower range are discarded randomly to balance the data in the training stage. Figure 3(b) indicates stiffness curves for the X-unit framework, and it shows that in most cases, the structure experiences buckling, which leads to various stiffness and nonlinearity. The stiffness distribution of the structures before and after buckling is depicted on the left and right sides of Figure 3(b), respectively, which clearly shows the structure represents higher stiffness in the first mode on average. The stiffness distribution of this framework is close to the normal distribution (skewed in the second state), which is proper to train our physics-informed model. Even though nonlinearity is not ideal, in some applications like soft robotics and energy absorption is required. To map the input parameters to the resulting stiffness curves efficiently, we train a PENN using the simulated data, following a series of preprocessing steps described below.

### 2.3. PENN

Several important preprocessing steps must be implemented when preparing the data to train a DNN. This network has a complex architecture, including multiple dense layers and a large output layer, and these preprocessing steps will help optimise how well the model performs. The initial dataset contains around 9,000 (as outlined above, 4000 for the F-unit and 5000 simulation data for the X-unit framework) time series outputs from Simulink that represent different configurations of the frameworks. The first step is to standardise the sample rates of all the time series data through a process called resampling. This ensures uniformity and mitigates potential differences in temporal resolution from the variable sampling steps of Simulink’s solver. After resampling, down sampling is performed on the stress-stress curve to lessen the computational workload without jeopardising important information. To ensure that valuable information of the curve is saved, the power spectrum of the signals is analysed to confirm that aliasing does not occur. From the thousands of samples in each stiffness curve, only 50 samples are saved to train the PENN, based on a trade-off between model accuracy and computational efficiency. We tested sampling resolutions of 25, 50, 75, and 100 points. While 100 points offered slightly better accuracy, 50 points provided a comparable performance with significantly reduced training time. It is important to use down sampling rates that keep essential features while removing excess data, preserving the integrity of the time series information. Lastly, normalisation is applied to scale the data within a consistent range. This aids model training convergence and improves generalisation. While the simulations are deterministic, PENN is used to learn complex nonlinear mappings – especially around buckling – rather than to model randomness.

Here, an 80:20 ratio is used to divide the data into training and test subsets. This allocates enough data for model learning while retaining a sizable portion for robust evaluation. This partitioning is conducted through the cross-validation methods that k-fold is performed here, which can improve reliability when assessing the model. K-fold repeatedly partitions the dataset into training and testing sets, and this iterative process provides insights into how well the model generalises across different data samples. We utilise a 5-fold validation method (80% for training and 20% for testing) to make sure that the model is not biased and provides more reliable performance evaluations. Various methods are used to evaluate a PENN and based on the task involving various feature input and predicting the stress–strain curve (including 50 points on the curve with a unified sampling rate), we used root mean squared error (RMSE) to evaluate the model’s performance.

**Figure 4.**
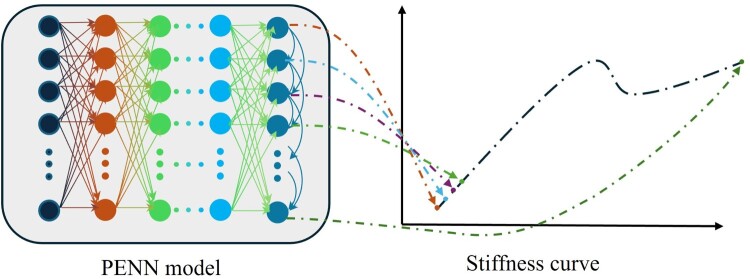
PENN architecture with the customised last layer to integrate the physical rules into the model.

Following this evaluation strategy, we detail the architecture of the PENN model designed to incorporate domain-specific physical rules into its learning framework. The model’s architecture includes a feature input layer that is defined based on the input data dimensions, excluding dimensions related to our frameworks. Initial differences between PENN predicted values and simulation outcomes were discovered, related to the neural network's performance. These were addressed systematically by fine-tuning the neural network's parameters, such as the architecture, learning rate, and loss function, to improve the model's prediction accuracy. This is followed by four fully connected layers with rectified linear unit (ReLU) activation. This architecture is selected empirically by evaluating several layer configurations and monitoring validation errors. The first layer has 128 nodes, then 256 nodes for the second layer, and 512 nodes for the third and fourth layers. The final fully connected layer contains 50 nodes matching the dimensions of the stiffness curve. Physical enhancement in the PENN model is introduced through both the architecture and the loss function to embed known mechanical behaviours. First, a customised layer is added to the architecture to integrate physics knowledge from the mechanical structure into the ML model. In this layer, the value of each node represents one of the 50 samples on the stiffness curve. To add the spatial dependency that is present in the stiffness curve, each node has an input from the upper node that represents the preceding sample. To add the changing rate information (which is the stiffness of the structure) to the layer, the penultimate node is connected; therefore, the PENN model can learn the local stable stiffness to predict the next sample with more precision (Figure 4). This network configuration, mimicking the temporal and physical dependency of mechanical systems, allows the model to discover complex relationships between input features and the target output, supporting the prediction of lattice stiffness values.

Secondly, the loss function incorporates two physics-based constraints. The trend_loss_ term mathematically enforces the physical condition of positive stiffness during stable loading. This penalises any portion of the predicted stiffness curve where force decreases with increasing strain – violating basic stability in elastic behaviour. In Equation (6), $y_{i}$ is the output of the *i*^th^ node of the model which represents i­^th^ sample of the curve. Therefore, the formula implies that when the one sample $\left(y_{i}\right)$ is bigger than the next sample ($y_{i+1}$), an extra cost is added to the total loss of the model. Furthermore, a buckling loss­ (reflects the physical behaviour of snap-through buckling, where a sudden decrease in stiffness occurs over a localised region) term is defined to improve the PENN in learning the snapping effect more effectively. In this term, the average of the decreasing trend is calculated only if that local downward trend is more than a margin (a coefficient) to avoid small amplitude noises. If the downward trend is lower than the buckling coefficient ($C_{\mathrm{buckling}}$), an extra loss value is added to the total loss function. This additional term enhances the utilised PENN performance (the performance is compared to a normal DNN in Supplementary Figure 3 to Supplementary Figure 6). Together, customised NN layer and customised loss function components encode the physical behaviours of stiffness continuity and buckling-induced transitions into the neural network, guiding learning in a way that respects the mechanical context of the problem.

$$\begin{array}{rl} & \mathrm{Tren}d_{\mathrm{loss}}\;=\;\overset{49}{\underset{i=1}{\sum }}\max(0,y_{i}-y_{i+1}{)}^{2}\mathrm{Bucklin}g_{\mathrm{loss}}\\ & =\max(0,C_{\mathrm{buckling}}-\;\mathrm{mean}[\;(y_{i}-y_{i+1})\;\mathrm{IF}\;(y_{i}-y_{i+1})>a\;]{)}^{2} \end{array}$$

(6) $$\mathrm{Tota}l_{\mathrm{loss}}=\mathrm{MS}E_{\mathrm{loss}}+\mathrm{tren}d_{\mathrm{loss}}+\mathrm{Bucklin}g_{\mathrm{loss}}$$

**Figure 5.**
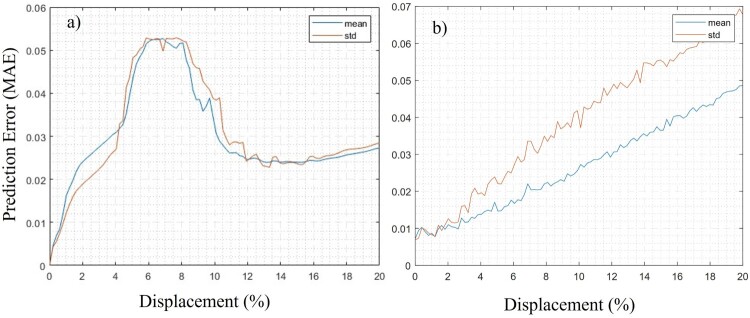
Mean and standard deviation of the error (mean absolute error (MAE)) of the trained PENN (a) for X-cell simulation results and (b) for F-cell structures.

Stochastic gradient descent with momentum (SGDM) optimisation is used to train the network along with a specified mini-batch size. Model performance is monitored on a validation dataset during training. This network structure is tailored to adequately capture the underlying patterns in the input data and generate precise predictions of framework stiffness values. Twenty percent of the data from Simulink is reserved for testing the trained model, as mentioned before. After training the model (the training error is depicted in Supplementary Figure 3 and Supplementary Figure 5 in the supplementary document), its performance is tested on the test data. Figure 5 shows the average error and its standard deviation for the proposed structure. In Figure 5(a), the error for the X-cell structure is less than 0.03, but it exceeds this line in the range of 4% to 12% strain. The buckling in various configurations happens in this range, which is harder to recall by the NN model and leads to more errors in its performance. The maximum of the mean and standard deviation of the error of the DNN in this region have decreased by 25% and 43%, respectively. Additionally, an ablation study was performed to assess the buckling loss term’s impact. Without it, the MAE in the buckling region was 0.039; with it, the MAE decreased to 0.032, a ∼18% improvement, confirming its role in enhancing buckling prediction accuracy. While, the PENN model was trained on synthetic ROM data for efficiency, loss function is designed with physical rules to avoid overfitting to simulation noises, and the result is validated with experimental data. In Figure 5(b), the error of the F-cell structure is illustrated, and it is higher than the error for other structures, despite its linear behaviour. While this range of error remains acceptable for this application, the error comes from the relatively smaller size of the model that is used for the F-cell structure. This model has 4 layers with 64, 128, 50 nodes, and the last customised layer that is explained above. The smaller model is designed to avoid overfitting and decrease the computational cost. The error of the PENN in this study is within an acceptable range and is comparable to other studies, which have reported MAE values of 0.31[34], 4.3 × 10−3 [29], and a reconstruction error of 0.3% [19]. Regularisation methods such as dropout and L2 weight decay were tested in preliminary trials but did not significantly improve performance, so we opted for simpler architectures with validation-based performance monitoring instead. All models were trained with early stopping based on validation loss and evaluated using separate test data to ensure generalisation.

### 2.4. Optimisation

In this study, the GA was used to iteratively improve lattice parameters based on natural selection and variation. Initially, a population of randomly generated potential parameter configurations was created, representing diverse structural characteristics. The potential parameter configurations were input to the PENN, which trained to predict lattice stiffness curves from parameters. Fitness scores were calculated by comparing predicted ($p_{i}$) to desired ($d_{i}$) stiffness curves with MAE formula in Equation (7), where the desired curve was randomly selected, and it can be set based on researchers’ need to design various structures. The difference quantified the fitness for each parameter configuration in the genetic algorithm population. The GA framework iteratively optimised the parameter configurations across generations using selection, crossover, and mutation operations. This minimised the difference between predicted stiffness curves from the neural network (NN) and the desired stiffness curves.

(7) $$\mathrm{MAE}=\frac{1}{N}\Sigma (\left|\;p_{i}-d_{i}\right|)$$

Upon completing the GA optimisation process, the final optimised parameter configuration underwent thorough evaluation using simulation techniques, especially within Simulink. The optimised lattice structure was implemented in Simulink to simulate behaviour under different scenarios. The key performance metric, mechanical response, was evaluated through simulation to determine the effectiveness of the optimisation process in enhancing the lattice's overall performance. Additionally, comparisons were made between the optimised lattice and the desired stiffness curve to measure improvements from the GA-based parameter optimisation. By integrating GA optimisation, PENN modelling and simulation, this study provides an all-encompassing method for advancing lattice structure design and performance optimisation. Our PENN runs in under a second, highlighting its computational efficiency versus the simulation solution which is 275 s (4.5 mins) on average. This major reduction in runtime underscores PENNs’ ability to lower computational complexity, providing a promising way to speed up optimisation processes and quicken engineering analyses.

**Figure 6.**
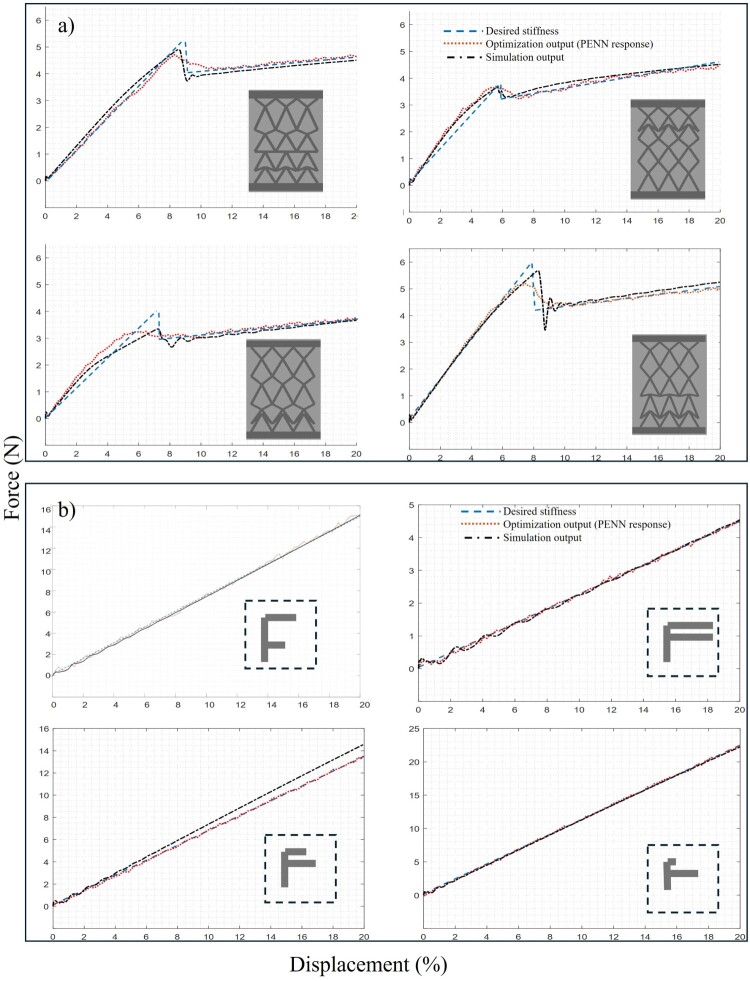
Comparison of the desired stiffness curve and the optimisation process output, validated by simulation (a) four samples of the X-cell framework and (b) four samples of the F-cells utilised in the framework.

The GA algorithm is implemented based on *gamultiobj* function on Matlab with the following parameter: 50 population, 100 generations, 80% crossover, adaptive mutation, tournament selection, stopping at function tolerance or max generations. The result of the GA search is depicted in Figure 6. In each figure, the desired stiffness curve (dashed blue line) is compared to the output of the optimisation process (dotted red line). Then, the optimised structure is simulated in Simulink to evaluate the optimisation process, and the simulated structures’ picture is added to each figure. In Figure 6(a), four samples of the X-cell structure are depicted, that buckling happens at various points and they have various stiffnesses before and after buckling. As is shown, the simulation results show the GA’s output configuration follows the desired curve with negligible error. The same process is repeated for the F-cell framework based on its linear response. The final simulation model response has less error regarding the desired stiffness, which is shown in Figure 6(b). Overall, the average error for all the samples including, F-cells and X-cells, doesn’t exceed 0.1, which was set as the error margin. After identifying the optimal design parameters through GA and PENN modelling, the next step is to physically realise and validate the structure via 3D printing.

### 2.5. Fabrication

In this study, an innovative methodology linking GA, PENN, and 3D printing was applied to create an optimised metamaterial structure on one platform. The optimised parameters from the GA were used to model the design precisely in Fusion 360, confirming its desired target performance. The digital model was then sliced into printable layers using Cura slicing software, carefully adjusting settings suited to the properties of TPU filament and the abilities of the Snapmaker 2 3D printer. Special attention was paid to Cura's slicing settings to account for the specific properties of the TPU filament diameter of 1.75 mm and ensure high print quality and structural strength. Due to some differences between the results of the 3D printing process, fabrication parameters such as extrusion temperature, printing speed, and retraction settings were optimised to minimise material inconsistencies and improve structural reliability. These parameters were held constant across all prints to maintain comparability between samples. This procedure was repeated until the algorithm displayed effective performance, with small differences between simulation and experimental results, so proving the robustness of the proposed method.

**Figure 7.**
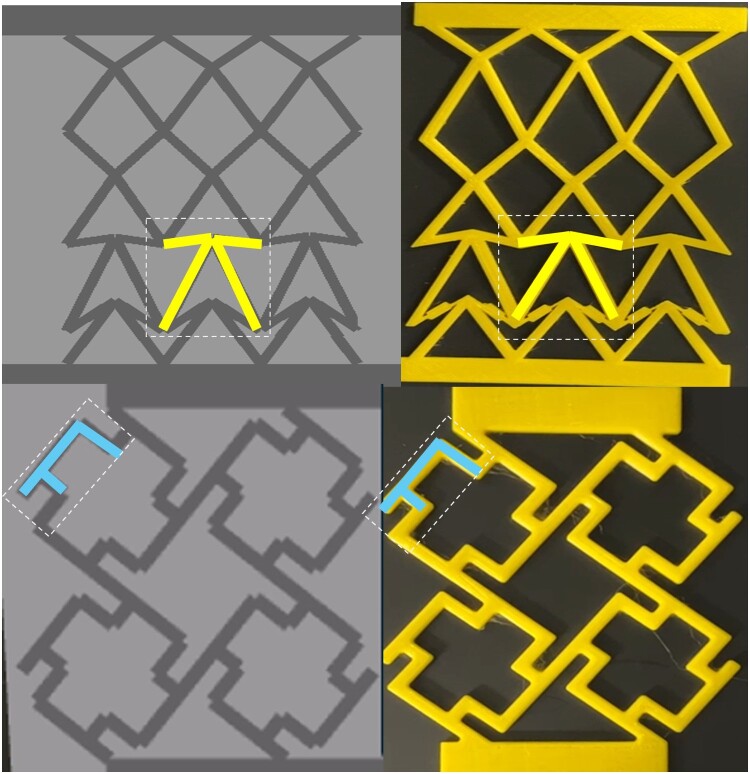
Simulated and fabricated geometry of the optimised structure for predefined stiffness (top: X-cell framework, down: F-cell framework with a highlighted cell).

Optimal extrusion and adhesion of the elastic filament required a printing temperature of 205°C and a heated bed temperature of 55°C. To mitigate the risk of printing unwanted material such as stringing or leaking, a conservative printing speed of 5 mm/s and a retraction speed of 60 mm/s were chosen, providing ample time for the TPU material to solidify between successive layers. All samples were printed horizontally with layers aligned to the loading direction to ensure consistent mechanical performance. Inter-layer bonding was optimised via controlled extrusion temperature and speed, and no post-processing steps were applied. Figure 7 depicts the printed structures for two samples of the F-unit and X-unit framework. (videos of the tests – included simulations and experiments – are provided in Supplementary Video 1–4). As shown in Figure 7, those links that are going through snapping in the X-unit framework were narrowed near joints (thickness to 1 mm over 1–2 mm) to match simulation joints which are idealised joints in just one points.

## 3. Results and discussions

**Figure 8.**
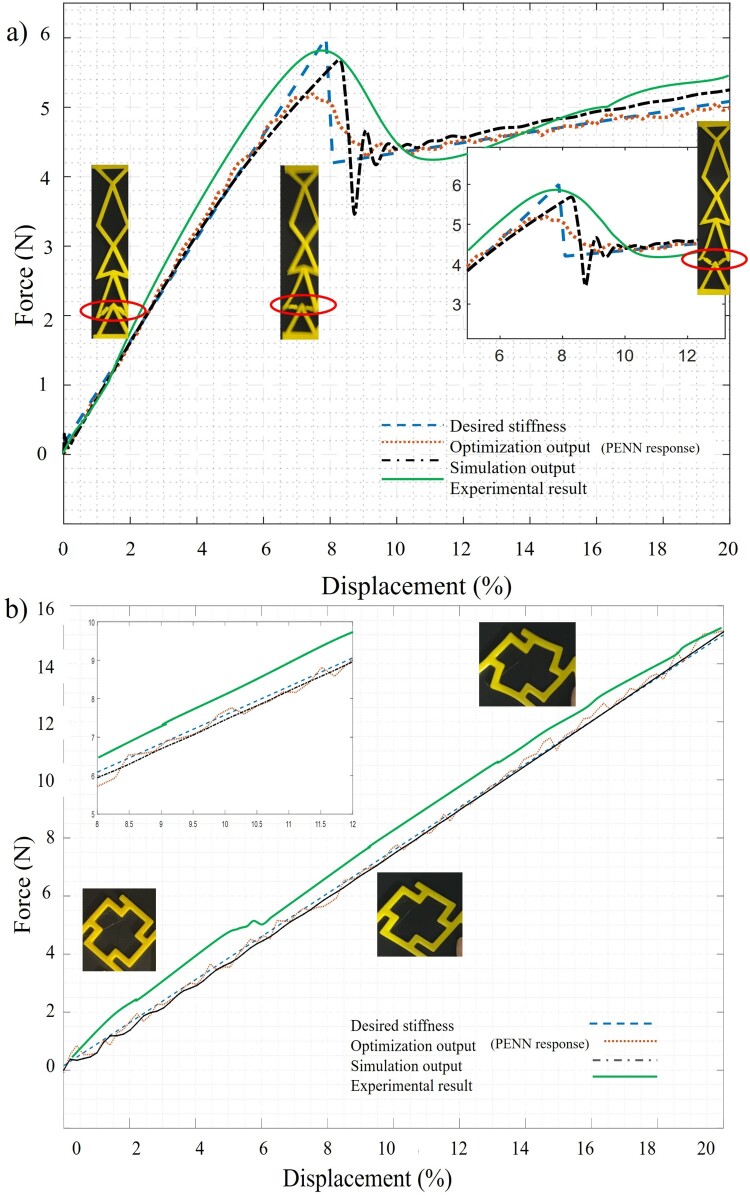
Comparison of the desired stiffness with the output of the PENN, simulation, and experimental results (a) Stiffness of the X-unit metamaterial (b) Stiffness of the F-unit metamaterial.

Compared to traditional ROM-based optimisation, which takes approximately 275 s per evaluation in MATLAB in the mentioned condition, the PENN predicts stiffness curves in under one second (running the PENN takes 0.011sec in average). For an optimisation run with 1000 evaluations, this translates to a reduction from ∼76.4 h to ∼11sec. This efficiency enables rapid inverse design iterations. While this study focuses on two representative unit-cell geometries and one material system (TPU), the framework is extensible. Different geometries can be encoded through parameterisation, and the model can be retrained or fine-tuned for other base materials or boundary conditions. However, generalisation across vastly different materials or nonlinear behaviours not present in the training data may require domain-specific retraining or the inclusion of material descriptors as input features. The mechanical tests on the 3D-printed structures (including X-unit and F-unit structures) revealed promising findings seen in their stress–strain graphs (Figure 8). At first, under lighter loads, the X-unit designed structures showed high rigidity, pointing to its proficiency, to support forces effectively.

As the load increased further, a snap through buckling response developed. Parts of the structure underwent snapping, causing an abrupt decrease in rigidity, while other portions maintained their form. It is noteworthy that in both states of stiffness, the stress–strain relation remains approximately linear. The notable stiffness initially detected can be connected to the organised alignment of components within the framework, providing rigidity against external forces. Snap-through buckling in the X-unit structure triggers a sudden configuration shift, reducing rigidity. This is modelled via flexible beams and nonlinear solvers in Simscape and observed experimentally as a stress–strain drop. The nonlinearity arises from geometric reconfiguration, which alters the load path and decreases effective stiffness during the transition from high to low stiffness phases. On the other hand, the F-unit structure remains linear and holds its stiffness throughout the full testing process. This difference in performance indicates an involved interplay of factors inside the metamaterial design, including its geometric setups. The F-unit framework provides tuneable linear stiffness and adjustable Poisson’s ratio, while the X-unit can exhibit responses ranging from nearly linear to highly nonlinear bistable behaviour depending on its configuration. This range, achievable within two-unit types, demonstrates the generality and adaptability of the proposed inverse design approach using PENN. Although the current structure includes a limited number of units, it demonstrates geometry-driven nonlinear behaviour – such as bistability and stiffness tuning – which are defining traits of metamaterials. This study serves as a proof-of-concept on the snap-through design of such structures while the future work will extend the approach to larger periodic arrays for scalable applications.

Differences between experimental and simulation testing can originate from several aspects intrinsic to both methods. A reason for these variations can arises from the performance of the NN in the buckling region. Several steps were included in this study that improved the performance of the algorithm in this challenging region, but further improvements can be made. For example, applying a nonuniform sampling rate to generate more data points specifically from the buckling region could help the PENN to capture the highly nonlinear behaviour of our data, which remains a challenge for the network. One clear restriction in simulations is the simplification of elaborate designs such as mechanical metamaterials, which may not completely represent real-world performance. For example, portraying the joints between structure’s links as single points can simplify the real behaviour, disregarding intricacies in load allocation and stress distribution. This simplification reduces simulation complexity and facilitates the generation of large-scale datasets. It focuses on the dominant bending and buckling responses, which drive global stiffness trends. Nonetheless, adding rotational compliance or hinge friction could improve local accuracy. In some designs, the thickness of the links has been modified around the structure’s joint to fabricate the structure with similar features as the simulated structure.

Additionally, the nonlinear nature of TPU presents difficulties in precisely predicting its mechanical reaction under large extensions, while it has been assumed linear in the simulation. This matter can be addressed by defining the non-linear stiffness of the material in the simulation section. Additionally, imperfections inherent in TPU printing, for example, inconsistencies in layer adhesion or voids inside the material, can further widen gaps between simulation and real testing outcomes. As it was mentioned before, one of the main differences is the joint formation; therefore, some of them are edited. To keep the changes minimum, only those links with obtuse angle (where buckling happens in the structure) in X-cell frameworks (Figure 1) are modified. The links are narrowed at both ends, which is shown in Figure 7 to make the simulated joints and the printed joints act similarly. These joint adjustments aligned the model with the simulation, with stiffness deviations under 5%. This study intentionally used TPU to focus on reliably capturing buckling and snap-through behaviour in a flexible, printable medium. While recent work [35,36] has explored tougher architectures emphasising sound absorption and elastic resilience, our approach complements these by targeting the inverse design of bistable or snapping metamaterials. Future work could combine these directions by incorporating tougher materials and resilient architectures with tuneable nonlinear responses using the proposed design framework. While the current work focuses on TPU to ensure feasibility with accessible 3D printing, materials with higher damping (e.g. viscoelastic polymers) or greater yield strength could be considered for applications like impact mitigation or durable structural elements. A strategic material selection process could thus complement structural optimisation to fully exploit metamaterial design potential. Although this study explores single-scale buckling behaviour, the proposed framework lays the groundwork for future research into hierarchical and multi-scale metamaterial systems.

Addressing these issues may necessitate further investigation with more accurate models. Firstly, refining simulation techniques to encompass intricate aspects of the metamaterial's architecture, for example, multi-point joints and diverse materials, can augment accuracy. Sophisticated computational methods, including FEA, coupled with testing, offer promising potential to heighten simulation precision. Moreover, technological developments in 3D printing involving updated material formulations and parameters could diminish imperfections, strengthening constitution and functionality. Further enhancing properties and decreasing inconsistency between virtual modelling and trials demands thorough quality measures during printing and post-refinement, like annealing. Beyond experimental validation, the versatility of the proposed metamaterial designs opens pathways for a range of real-world applications, as illustrated in the following examples.

**Figure 9.**
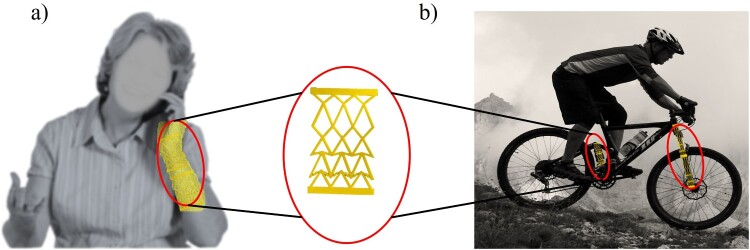
Designed metamaterial application. (a) Wearable hand tremor attenuator. (b) Bicycle suspension system.

The metamaterials show promise for various applications that with the proposed method the structure and functionality can be customised. Two notable examples include wearable hand tremor dampers and suspension systems for vehicles (Figure 9). A tremor-reducing device could harness the engineered metamaterial's ability to alternately change between flexible (after buckling in the metamaterial that shows lower stiffness) and rigid (before the snapping effect that the framework shows higher stiffness) configurations, and by transitioning between these states, it could optimise the user experience by providing more flexibility or resistance based on the users’ desires. Individuals with tremor experience vibration in their hands in various situations, depending on their condition (such as Parkinson’s disease or essential tremor). Tremor force is less than voluntary force, therefore the structure stays at less strain rate and acts in a high stiffness phase (Figure 3), when individuals apply higher force (voluntary motion), the structure’s strain is higher (after buckling) causing the structure to respond in lower stiffness (second phase in Figure 3). Therefore, these wearable devices can be flexible in the absence of tremors, giving the subject more comfort. The proposed application is not just limited to tremor mitigation devices. But it can also be utilised in rehabilitation or physical training applications. The stiffness of this metamaterial in both states is customisable and could be designed based on the individual’s conditions. This ability and its other features, such as being lightweight and having a low volume, make this structure a great candidate for wearable devices.

Suspension systems for vehicles are quite important across different driving situations. They help smooth out the ride to improve passenger comfort while maintaining stability and control, no matter the road conditions. These systems can adjust to suit varying needs. A variable stiffness metamaterial structure could be built into suspension parts of bikes like shock absorbers or springs. This would allow the mechanical structure to alter its rigidity. The structure retains a higher stiffness (first phase, Figure 3) for the best possible vehicle support and stability when driving on smooth roads. However, buckling occurs when it encounters bumps, potholes, or uneven roadways because of the heavy load that leads to higher strain, which reduces the rigidity (transition to lower stiffness phase, Figure 3). By doing this, the suspension system's total stiffness is decreased, improving its ability to manage uneven terrain. This ability to adapt allows the bike to better absorb and reduce impact forces, improving comfort for the rider while lessening vibrations. Additionally, the ability to dynamically adjust stiffness based on riding conditions enhances bike agility and handling, providing a smoother, more controlled experience for the rider.

The proposed framework allows stiffness customisation by defining an application-specific target curve – such as user-specific hand dynamics for tremor damping or suspension performance profiles for vehicles – as the objective for the genetic algorithm. The PENN model then rapidly identifies structural parameters that reproduce the desired behaviour, enabling tailored metamaterial designs without high simulation costs. From a manufacturing perspective, consistent performance may be challenged by variations in filament quality, printer calibration, or environmental conditions. To address this, quality assurance methods such as layer-wise monitoring, process standardisation, and post-print validation can be applied. While our method is well-suited for personalised or small-batch applications, future work will focus on ensuring reproducibility and investigating scalability for broader manufacturing.

## 4. Conclusions

This study established and verified a unique method for the inverse design of 3D-printed mechanical metamaterial structures with the desired behaviour by combining machine learning techniques. The basis of the flexible body simulation in MATLAB/Simscape was first established using the mathematical model of the structure. The enhanced NN was then trained using a large dataset that was obtained from 9000 stress–strain simulations of two different metamaterial cell designs. This training made it easier to create a thorough relationship between the design parameters and the mechanical reactions of metamaterials, such as the range of the stress–strain responses and nonlinear snap through buckling.

A customised NN was designed by integrating the physical rules governing the stiffness of metamaterials. The PENN was tailored in both architecture and loss function to enhance its training efficiency for learning the buckling effect. The loss function was updated with two new terms to enhance the model’s efficiency in predicting the stiffness curve, especially the nonlinear section (buckling effect) that was challenging for DNN. The effectiveness of the PENN was demonstrated by their ability to predict stiffness curves rapidly and with reasonable accuracy. Compared with the simulation methods, the proposed method is slightly less accurate which reflects a trade-off between computational cost and precision.

Subsequently, an inverse design methodology was implemented using genetic algorithms. This method proved capable of optimising metamaterial designs to effectively satisfy predefined mechanical responses effectively. The optimised designs were then fabricated using 3D printing techniques, specifically employing TPU to experimentally validate the strain–stress responses under tensile force. The experimental results were observed to closely aligned with both the simulations and predictions made by the PENNs, thereby confirming the practical applicability of the proposed design paradigm. Challenges such as the simplification of complex designs and the linear approximation of Young’s modulus of the TPU material were acknowledged as limitations that could impact the fidelity of simulations and experimental outputs. To overcome these obstacles, future research will incorporate more thorough joint mechanics modelling and delve deeper into the nonlinear properties of materials. Furthermore, it is recommended that more research be conducted to convert the existing 2D metamaterial designs into 3D structures. This might improve functionality and increase the range of applications in industries including adaptive robots, wearable technology, and automotive safety systems.

Overall, the integrated approach presented in this study not only validates the feasibility of using customised NNs and GAs for the design and fabrication of mechanical metamaterials but also demonstrates the efficiency of conducting the entire workflow within one computational environment. The method opens up new avenues for the development of intelligent material systems capable of complex, programmable responses. We anticipate that this work will pave the way for the engineering of functional metamaterial patterns, enabling the development of structures with shape-shifting and variable stiffness. By collecting reliable simulation results and altering the architecture of the PENN, the proposed method can be utilised for developing materials in various shapes such as beams, sheets, while being committed to the assumption on this study including the negligible strain in the material. With the suggested framework, designers can identify the best geometric factors given a set of limits and nonlinear requirements.

## Supplementary Material

Supplemental Material

## Data Availability

The data that support the findings of this study are available from the corresponding author, A. Zolfagharian, upon reasonable request. A live link to dataset could be found as follows: https://drive.mathworks.com/sharing/3f7d1658-bf86-42d9-a1f7-8f49a81b8faa.
