# Supplementary material for: Inverse design of adaptive flexible structures using physical-enhanced neural network
Source: Virtual Phys Prototyp. 2025 Jul 18;20(1):e2530732. doi: 10.1080/17452759.2025.2530732 (PMC12502832; doi:10.1080/17452759.2025.2530732)
Supplement: Supplemental Material [file NVPP_A_2530732_SM8449.docx]

Supplementary Documents

**Flexible Mechanical Metamaterials Rapid Design and Prototyping Using Computationally Efficient Reduced Order Model**

Moslem Mohammadi^1^, Abbas Z. Kouzani^1^, Mahdi Bodaghi^2^, Ali Zolfagharian ^1*^

^1^ School of Engineering, Deakin University, 3216, Australia

^2^ Department of Engineering, School of Science and Technology, Nottingham Trent University, Nottingham NG11 8NS, UK

*S1. Mathematical model*

In this section the mathematical model is developed for the used frameworks in the paper (Supplementary Figure 1). Utilizing finite element method, the structure is divided to various sections and then is analysed under external force. The mathematical model is started based on motion equation:

$M\ddot{u}_{d}+L\dot{u}_{d}+Ku_{d}=f$ (1)

$\left[ \begin{matrix} M_{bb} & M_{bi} \\ M_{ib} & M_{ii} \end{matrix} \right]\left[ \begin{aligned} \ddot{u}_{b} \\ \ddot{u}_{i} \end{aligned} \right]+\left[ \begin{matrix} L_{bb} & L_{bi} \\ L_{ib} & L_{ii} \end{matrix} \right]\left[ \begin{matrix} \dot{u}_{b} \\ \dot{u}_{i} \end{matrix} \right]+\left[ \begin{matrix} K_{bb} & K_{bi} \\ K_{ib} & K_{ii} \end{matrix} \right]\left[ \begin{aligned} u_{b} \\ u_{i} \end{aligned} \right]=\left[ \begin{aligned} f_{b} \\ f_{i} \end{aligned} \right]$ (2)

The subscripts b and i refer to boundary and internal nodes respectively. Expressing the partitioned nodal degrees of freedom in terms of boundary and modal coordinates results in:

$\left[ \begin{aligned} u_{b} \\ u_{i} \end{aligned} \right]=H\left[ \begin{aligned} u_{b} \\ \eta\end{aligned} \right]$ (3)

where $\boldsymbol{\eta}$indicating the modal degrees of freedom. With some value in $\boldsymbol{u}_{b}$ specified in physical space and some in $\boldsymbol{\eta}$ vector defined in modal space, the degrees of freedom $\left[ \begin{aligned} \boldsymbol{u}_{b} \\ \boldsymbol{\eta} \end{aligned} \right]$ constitute a hybrid set. The matrix that links the two sets of coordinates is called the Craig-Bampton transformation, or **H**. The Craig-Bampton transformation matrix **H** contains **Ξ**, a set of constraint modes resulting from static condensation, and **Φ**, a set of fixed-boundary mode shapes.

$H=\left[ \Xi\phi\right]$ (4)

Limiting the modal coordinates to frequencies below a certain threshold excludes the lowest amplitude modes above that cutoff frequency and leads to the simplified equation:

$\left[ \begin{aligned} u_{b} \\ u_{i} \end{aligned} \right]\cong H\left[ \begin{aligned} u_{b} \\ \eta^{*} \end{aligned} \right]$ (5)

Limiting the modal coordinates based on a frequency cutoff removes the highest frequency modes, leaving only the truncated modal degrees of freedom, $\boldsymbol{\eta}^{*}$. Expressing the equation of motion using these reduced degrees of freedom results in the working model.

$\left[ \begin{matrix} \hat{M}_{bb} & \hat{M}_{bm} \\ \hat{M}_{mb} & \hat{M}_{mm} \end{matrix} \right]\left[ \begin{aligned} \ddot{u}_{b} \\ \ddot{\eta}^{*} \end{aligned} \right]+\left[ \begin{matrix} \hat{L}_{bb} & \hat{L}_{bm} \\ \hat{L}_{mb} & \hat{L}_{mm} \end{matrix} \right]\left[ \begin{matrix} \dot{u}_{b} \\ \dot{\eta}^{*} \end{matrix} \right]+\left[ \begin{matrix} \hat{K}_{bb} & \hat{K}_{bm} \\ \hat{K}_{mb} & \hat{K}_{mm} \end{matrix} \right]\left[ \begin{aligned} u_{b} \\ \eta^{*} \end{aligned} \right]=\left[ \begin{aligned} f_{b} \\ 0 \end{aligned} \right]$ (6)

The vibration modes of the body are indicated by the subscript m. The truncated mass matrix terms ${\hat{\boldsymbol{M}}}_{**}$, damping matrix ${\hat{\boldsymbol{L}}}_{**}$and stiffness matrix terms ${\hat{\boldsymbol{K}}}_{**}$represent the reduced mass matrix $\hat{\boldsymbol{M}}$, damping matrix $\hat{\boldsymbol{L}}$ and stiffness matrix $\hat{\boldsymbol{K}}$ respectively.

$\hat{K}=H^{T}KH$ and $\hat{L}=H^{T}LH$ and $\hat{M}=H^{T}MH$ (7)

It is assumed external forces act only on the boundary nodes, with no forces on internal nodes (f_i_ = 0). This yields the Craig-Bampton equation of motion using reduced matrices. Further processing of these reduced matrices provides the basis for determining the state-space representation matrices that is as follows:

$\dot{x}=Ax+Bu$ ,

$y=Cx+Du$ (8)

The states (x) in the finite element model representation are defined as the amplitudes factors of the different vibration modes (η) and the inputs (u) is represented as the motion of the boundary nodes ($\boldsymbol{u}_{b}$), and the outputs (y) is the internal forces and torques (-f_b_) that develop within the structure due to the deflections at the nodes.

$$\boldsymbol{x}=\left[ \begin{aligned} \boldsymbol{\eta}^{*} \\ {\dot{\boldsymbol{\eta}}}^{*} \end{aligned} \right]$$

$$\boldsymbol{u}\mathbf{=}\left[ \begin{aligned} \boldsymbol{u}_{\boldsymbol{b}} \\ {\dot{\boldsymbol{u}}}_{\boldsymbol{b}} \\ {\ddot{\boldsymbol{u}}}_{\boldsymbol{b}} \end{aligned} \right]$$

$\boldsymbol{y=-}\boldsymbol{f}_{\boldsymbol{b}}$ **(9)**

The reduced damping, mass, and stiffness matrices from the reduced finite-element model are transformed based on of the state-space matrices to provide:

$$A=\left[ \begin{matrix} O & I \\ {-\hat{M}}_{mm}^{-1}\hat{K}_{mm} & {-\hat{M}}_{mm}^{-1}\hat{L}_{mm} \end{matrix} \right]$$

$$B=\left[ \begin{matrix} O & O & O \\ {-\hat{M}}_{mm}^{-1}\hat{K}_{mb} & {-\hat{M}}_{mm}^{-1}\hat{L}_{mb} & {-\hat{M}}_{mm}^{-1}\hat{M}_{mb} \end{matrix} \right]$$

$$C=\left[ \begin{matrix} -(\hat{K}_{bm}-\hat{M}_{bm}\hat{M}_{mm}^{-1}\hat{K}_{mm}) & -(\hat{L}_{bm}-\hat{M}_{bm}\hat{M}_{mm}^{-1}\hat{L}_{mm}) \end{matrix} \right]$$

$D=\left[ \begin{matrix} -(\hat{K}_{bb}-\hat{M}_{bm}\hat{M}_{mm}^{-1}\hat{K}_{mb}) & -(\hat{L}_{bb}-\hat{M}_{bm}\hat{M}_{mm}^{-1}\hat{L}_{mb}) & -(\hat{M}_{bb}-\hat{M}_{bm}\hat{M}_{mm}^{-1}\hat{M}_{mb}) \end{matrix} \right]$ (10)


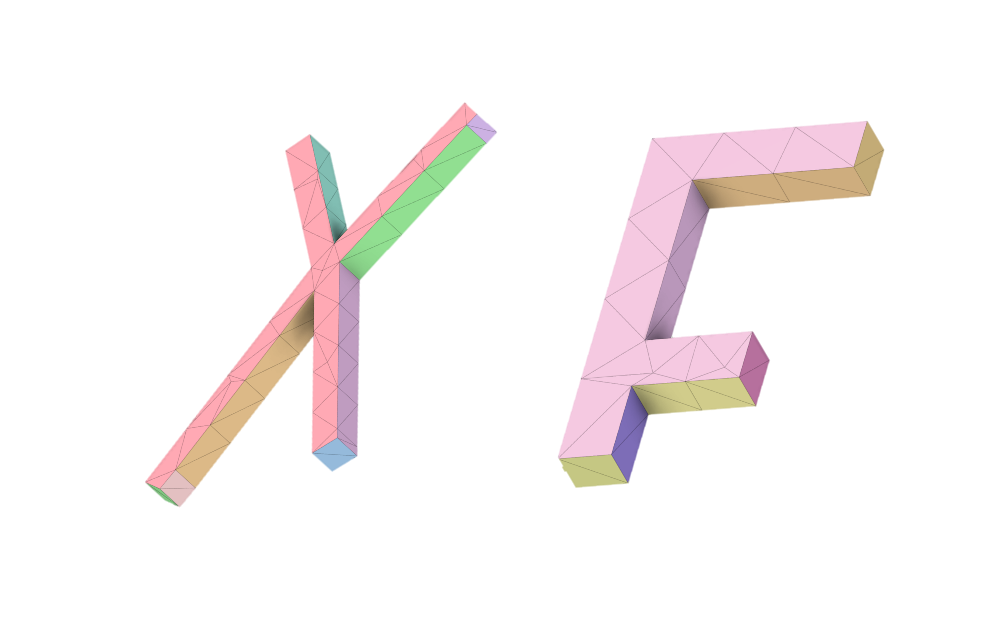


Supplementary Figure 1. Finite element polygons of the frameworks: X-cell (left) and F-cell (right).

*S2. Simulation*

The simulation of the structure is performed in MATLAB/Simscape based on flexible bodies. Each link in the structure is simulated with one rectangular flexible beam that its dimension is set randomly. The young’s modulus of the beams is set with a constant value in this simulation. Supplementary Figure 2 illustrates the setup of the stress-strain test on the structure and the simulation of the various configurations. The features of the structure are set randomly controlled by MATLAB script and run in parallel mode on a 12-core CPU. The set of the variable parameters for each framework is different. For the X-cell framework, the parameters set contains beams cross section (links height and their thickness), the beams length, the angles of the leg links and hand links that are defined by rigid transform blocks in Simulink. For the other structure, thickness and height of the beams are in the variable parameters that are set by the beams cross section. Also the beams length and the distance of the links of each other (head link to hand link) are the rest of the parameters that can be randomly valued and build and different structure with various stiffness. The stretching force is applied on a prismatic joint that is connected to both sides of the structure, then the displacement is read by the joint sensor and stored to be utilized in neural network training.

a)

b)

c)

d)


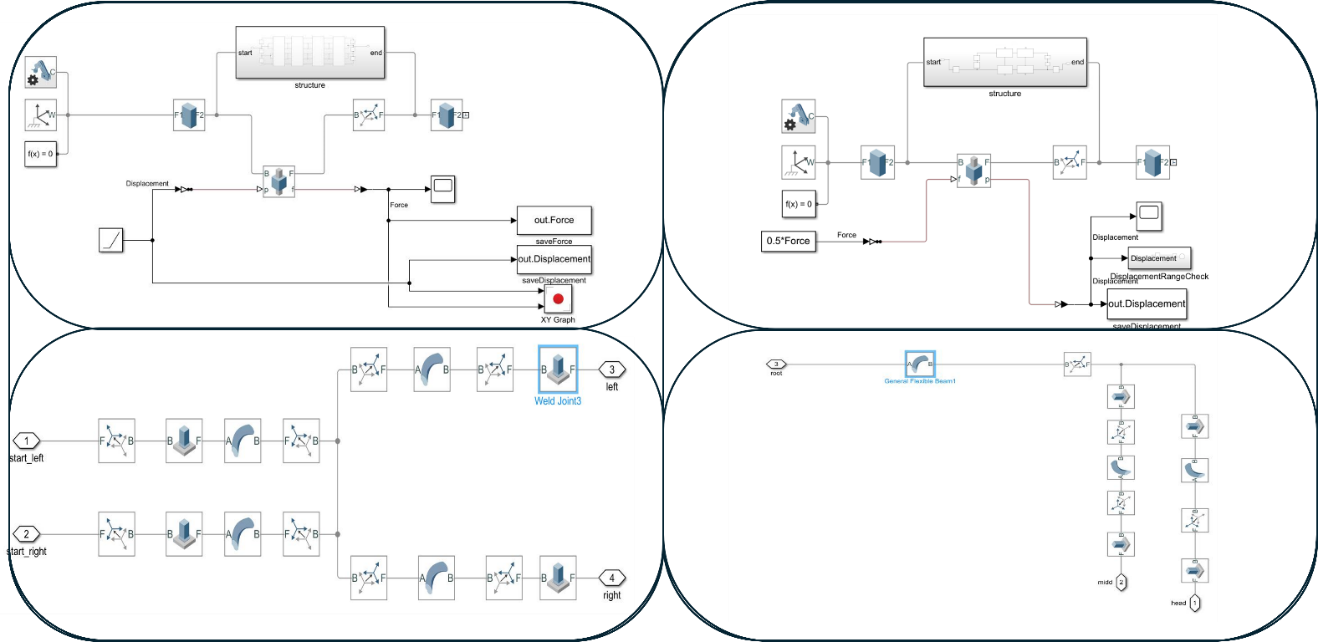


Supplementary Figure 2. simulation model of the frameworks a) The X-cell frameworks and b) F-cell frameworks simulated in Matlab. c) One single X-cell with four flexible beams and d) F-cell in Simulink environment with three flexible beams.

S3. PENN vs DNN

a)

b)


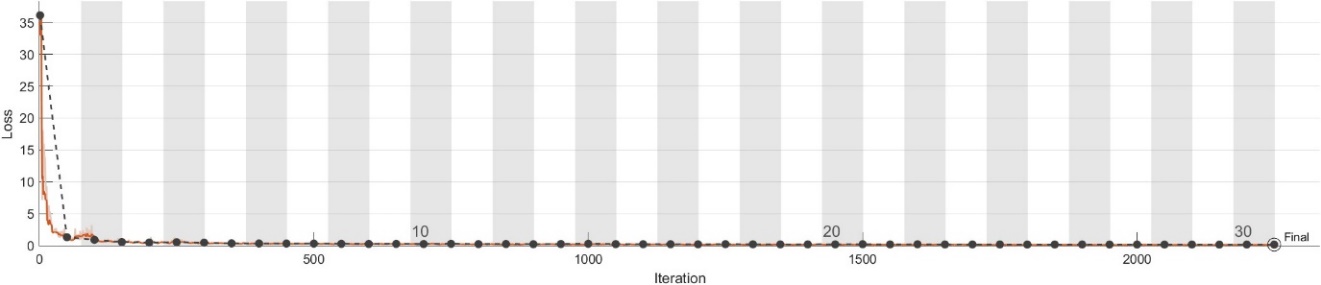

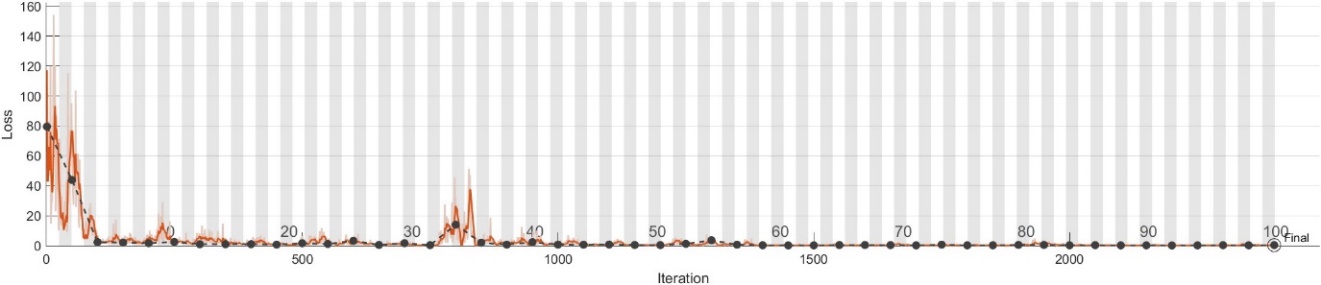


Training

Training(smoothed)

Validation

Supplementary Figure 3. Loss function output during the training for the a) DNN model and b) PINN model related to the F-cell structure.

In this section, the performance of the designed physics-informed neural network (PINN) is compared to deep neural network (DNN). Supplementary Figure 3 depicts the loss function for DNN and PINN models of the F-cell structure. The additional trend­_loss_ have made the values of the loss function bigger for the PINN model. Therefore, the error between the labels and the predicted values are calculated and its histogram and mean and standard deviation for each sample are depicted in Supplementary Figure 4. The vertical red line in Supplementary Figure 4.a and 4.b shows that 90 percent of the error margin. As it is presented in this figure, the overall error is reduced by utilising the PINN. Similar comparison is performed for PINN and DINN, trained on X-cell structure data. the results are shown in Supplementary Figure 5 and Supplementary Figure 6. These results proofs that by utilising the PINN and integrating physics rules in the architecture and loss function, the model can learn better with higher performance. By comparing Supplementary Figure 6.c and Supplementary Figure 6.d, it is clear that the error in the buckling region (4 to 12% stretched) is significantly decreased in PINN. The average of the error is decreased by 25% and the error in its standard deviation is decreased by 43%.


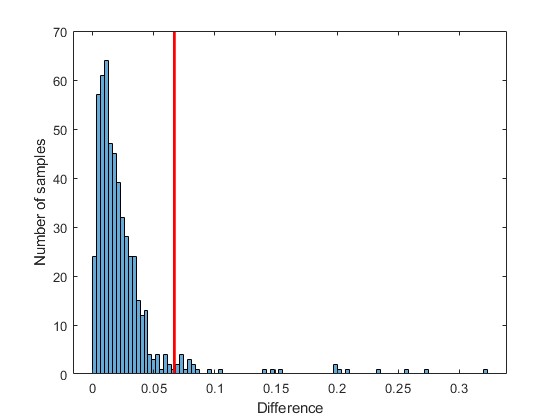

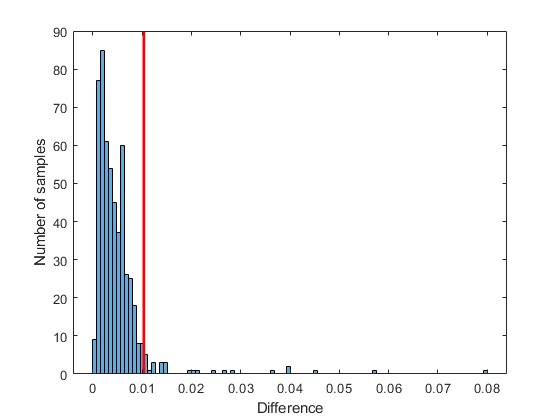

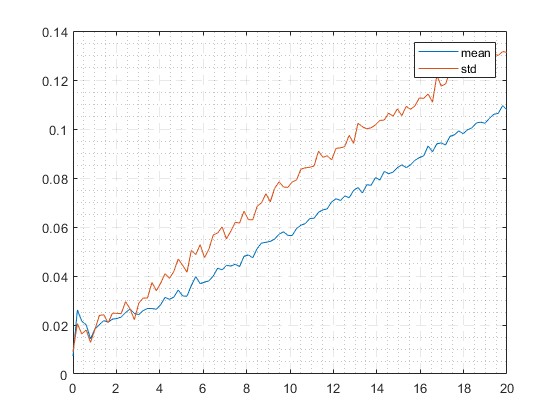

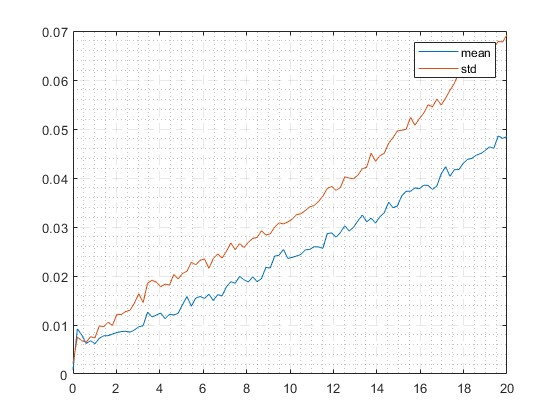


a)

b)

c)

d)

Error in force prediction (N)

Error in force prediction (N)

Displacement (%)

Displacement (%)

Supplementary Figure 4. Histogram of the error (mean squared error (MSE)) between labels and the output of the a) DNN model and b) PINN model. The vertical red line in (a) and (b) marks the 90th percentile of the data. c) The average and the standard deviation of the error (mean absolute error (MAE)) between the stiffness curve and the predicted curve for DNN model and d) PINN model.


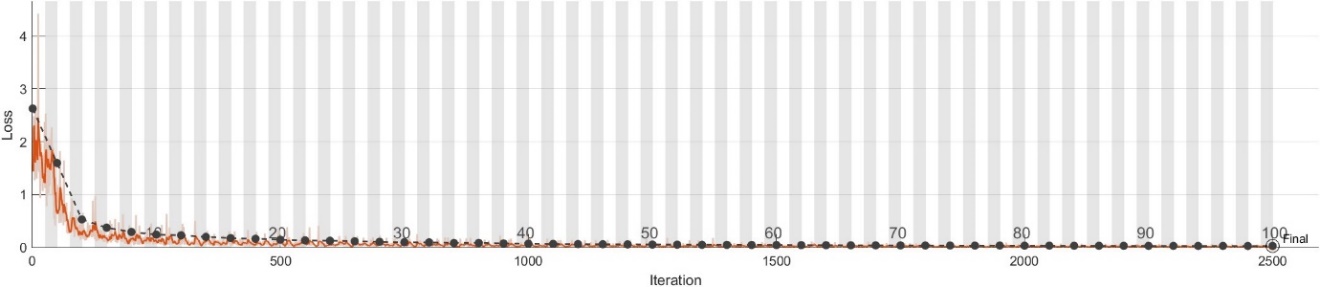

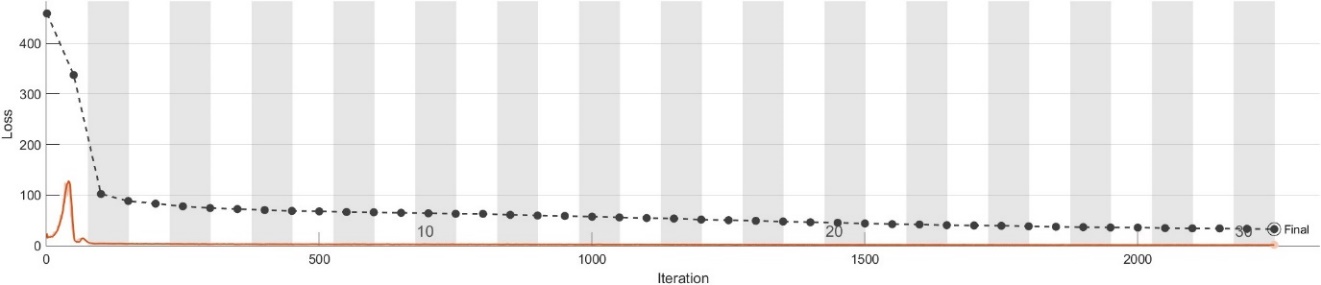


Training

Training(smoothed)

Validation

a)

b)

Supplementary Figure 5. Loss function output during the training for the a) DNN model and b) PINN model related to the F-cell structure.


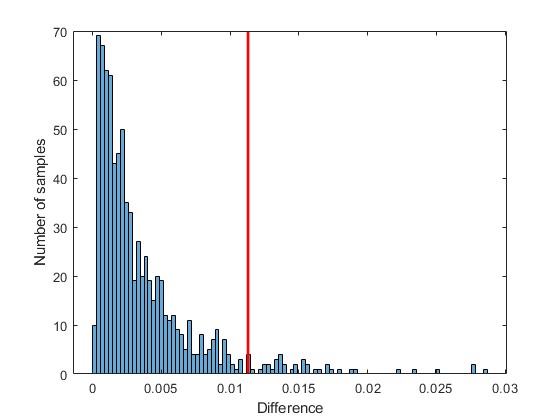

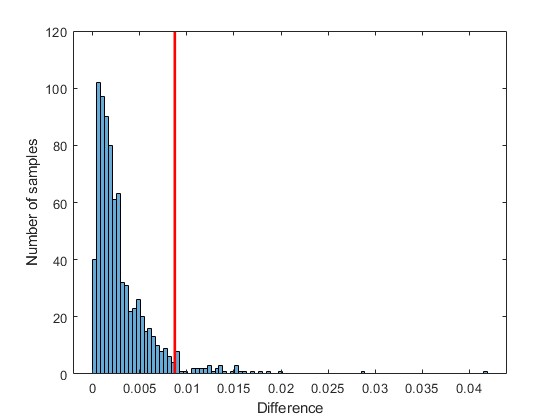

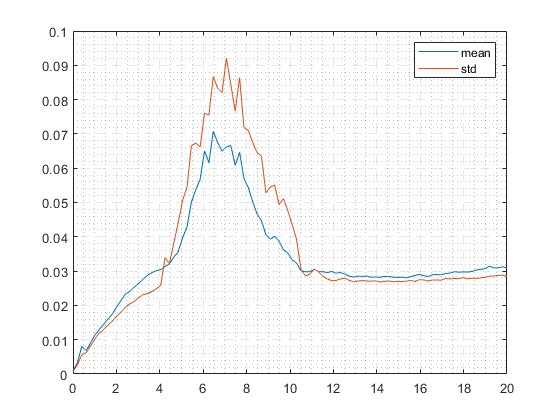

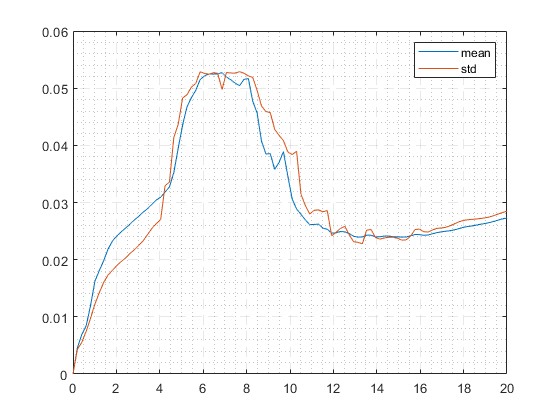


a)

b)

c)

d)

Displacement (%)

Displacement (%)

Error in force prediction (N)

Error in force prediction (N)

Supplementary Figure 6. Histogram of the error (mean squared error (MSE)) between labels and the output of the a) DNN model and b) PINN model. The vertical red line in (a) and (b) marks the 90th percentile of the data. c) The average and the standard deviation of the error (mean absolute error (MAE)) between the stiffness curve and the predicted curve for DNN model and d) PINN model.

*S4. Videos*

The *Supplementary Videos* show the experiment of the framework and their simulation.

Supplementary Video 1

<https://youtu.be/PerQyTpvK3E>

Supplementary Video 2

<https://youtu.be/8OYd1j_rA88>

Supplementary Video 3

<https://youtu.be/jspRruglN_I>

Supplementary Video 4

<https://youtu.be/AVu2gxwrdRY>
